# Supplementary material for: A Likelihood Approach for Real-Time Calibration of Stochastic Compartmental Epidemic Models
Source: PLoS Comput Biol. 2017 Jan 17;13(1):e1005257. doi: 10.1371/journal.pcbi.1005257 (PMC5240920; doi:10.1371/journal.pcbi.1005257)
Supplement: S1 File — (TAR.GZ) [file pcbi.1005257.s014.tar.gz › HSPH_Online-SI-Revision/EnKF07/n10-mild/n10-mild_EnKF2_table4.pdf]

1	newly 3 weeks spec	153.	94.68	{0., 303.}	85.9869	1.7887	newly 3 weeks cumul	425.	1210.94	{300., 1440.}	199.92	0.370234	newly 5 weeks spec	103.	61.53	{0., 151.}	89.5825	1.72132	newly 5 weeks cumul	653.	1346.81	{375., 1884.}	125.652	0.287051	newly 46 weeks cumul	1470.	1569.87	{482., 3225.}	75.2388	0.32606
2	newly 3 weeks spec	110.	245.47	{0., 797.}	245.918	1.56295	newly 3 weeks cumul	464.	2177.69	{172., 8128.}	398.588	0.435931	newly 5 weeks spec	57.	54.95	{0., 75.}	141.596	1.52676	newly 5 weeks cumul	610.	2350.61	{178., 8469.}	332.605	0.456035	newly 51 weeks cumul	742.	2400.4	{178., 8612.}	285.496	0.483952
3	newly 3 weeks spec	211.	284.95	{2., 889.}	146.204	0.958412	newly 3 weeks cumul	768.	1562.7	{49., 3126.}	179.654	0.576702	newly 5 weeks spec	97.	112.3	{0., 365.}	153.299	1.55994	newly 5 weeks cumul	973.	1868.09	{51., 4142.}	174.091	0.624911	newly 54 weeks cumul	1157.	1983.37	{51., 4397.}	158.822	0.65272
4	newly 3 weeks spec	113.	218.41	{0., 147.}	185.106	1.27496	newly 3 weeks cumul	452.	2283.05	{523., 7929.}	412.706	0.48205	newly 5 weeks spec	81.	69.81	{0., 92.}	125.691	1.40367	newly 5 weeks cumul	619.	2474.68	{569., 8348.}	309.683	0.401322	newly 49 weeks cumul	827.	2628.57	{574., 8348.}	238.55	0.37004
5	newly 3 weeks spec	93.	76.51	{1., 301.}	104.957	1.05277	newly 3 weeks cumul	276.	1361.48	{225., 3818.}	402.877	0.461797	newly 5 weeks spec	92.	14.45	{0., 54.}	86.3804	1.9192	newly 5 weeks cumul	472.	1405.45	{236., 4006.}	219.379	0.332239	newly 29 weeks cumul	1568.	1418.78	{238., 4044.}	95.1518	0.492693
6	newly 3 weeks spec	108.	98.88	{0., 115.}	133.907	1.50991	newly 3 weeks cumul	406.	862.89	{32., 2611.}	186.672	0.516601	newly 5 weeks spec	80.	56.69	{0., 64.}	127.638	1.57041	newly 5 weeks cumul	611.	996.86	{33., 2611.}	150.183	0.541457	newly 55 weeks cumul	829.	1124.71	{36., 2611.}	132.561	0.587549
7	newly 3 weeks spec	89.	64.9	{0., 116.}	111.528	1.75524	newly 3 weeks cumul	391.	1719.37	{57., 6889.}	395.471	0.54575	newly 5 weeks spec	87.	55.81	{0., 136.}	113.966	1.83021	newly 5 weeks cumul	552.	1834.6	{63., 6988.}	291.504	0.526215	newly 51 weeks cumul	801.	2184.98	{71., 8107.}	243.895	0.586764
8	newly 3 weeks spec	197.	185.94	{13., 237.}	113.675	0.645292	newly 3 weeks cumul	615.	1425.73	{254., 4988.}	177.494	0.338071	newly 5 weeks spec	164.	63.94	{0., 78.}	104.146	1.45977	newly 5 weeks cumul	920.	1609.95	{254., 5802.}	146.129	0.406888	newly 46 weeks cumul	2034.	1705.66	{254., 5894.}	95.4503	0.572264
9	newly 3 weeks spec	116.	277.61	{9., 1195.}	236.784	0.663496	newly 3 weeks cumul	592.	1300.32	{94., 3550.}	189.818	0.460441	newly 5 weeks spec	85.	97.47	{0., 132.}	155.094	1.18606	newly 5 weeks cumul	760.	1552.56	{114., 5891.}	185.758	0.505769	newly 50 weeks cumul	872.	1697.52	{114., 8164.}	182.289	0.531132
10	newly 3 weeks spec	91.	177.91	{0., 323.}	148.604	0.782733	newly 3 weeks cumul	345.	1787.62	{598., 3066.}	418.748	0.544863	newly 5 weeks spec	65.	25.86	{0., 45.}	78.3077	1.00217	newly 5 weeks cumul	482.	1879.57	{620., 3157.}	291.015	0.427351	newly 47 weeks cumul	1027.	1896.25	{625., 3248.}	111.941	0.244997
11	newly 3 weeks spec	165.	64.66	{2., 63.}	102.642	1.12688	newly 3 weeks cumul	495.	320.33	{110., 338.}	84.2485	0.47774	newly 5 weeks spec	56.	46.47	{0., 27.}	147.518	1.72788	newly 5 weeks cumul	648.	420.19	{139., 395.}	94.7731	0.557888	newly 52 weeks cumul	801.	600.93	{148., 403.}	112.331	0.631041
12	newly 3 weeks spec	53.	182.49	{8., 123.}	321.566	0.448384	newly 3 weeks cumul	224.	596.47	{76., 379.}	218.442	0.329237	newly 5 weeks spec	9.	122.52	{2., 68.}	1301.33	0.588899	newly 5 weeks cumul	264.	871.75	{103., 500.}	280.466	0.335559	newly 54 weeks cumul	276.	1191.08	{129., 1102.}	370.043	0.331239
13	newly 3 weeks spec	135.	152.65	{7., 122.}	108.2	0.539541	newly 3 weeks cumul	528.	1586.65	{378., 4829.}	221.956	0.333449	newly 5 weeks spec	51.	51.4	{0., 32.}	142.627	1.03038	newly 5 weeks cumul	676.	1730.28	{393., 5478.}	191.373	0.348394	newly 46 weeks cumul	774.	1774.8	{397., 5902.}	174.437	0.364127
14	newly 3 weeks spec	150.	142.3	{0., 291.}	109.12	1.329	newly 3 weeks cumul	578.	1139.88	{268., 3264.}	147.391	0.350528	newly 5 weeks spec	106.	33.25	{0., 108.}	83.7453	1.71516	newly 5 weeks cumul	857.	1240.53	{268., 3390.}	119.149	0.413068	newly 47 weeks cumul	1338.	1296.85	{269., 3411.}	90.63	0.474797
15	newly 3 weeks spec	208.	365.8	{0., 1296.}	191.106	1.38487	newly 3 weeks cumul	656.	2577.36	{446., 8408.}	314.942	0.377004	newly 5 weeks spec	192.	141.62	{0., 782.}	115.188	1.59192	newly 5 weeks cumul	1065.	2948.62	{505., 10 175.}	229.876	0.408447	newly 49 weeks cumul	2326.	3175.76	{531., 11 519.}	135.493	0.544892
16	newly 3 weeks spec	196.	415.87	{5., 1419.}	193.087	0.692806	newly 3 weeks cumul	619.	1862.34	{86., 7400.}	255.787	0.525864	newly 5 weeks spec	104.	140.35	{0., 443.}	136.817	0.938767	newly 5 weeks cumul	881.	2237.28	{114., 8630.}	213.737	0.525042	newly 50 weeks cumul	1128.	2573.98	{131., 9326.}	188.282	0.513722
17	newly 3 weeks spec	103.	295.16	{17., 1294.}	227.01	0.380805	newly 3 weeks cumul	286.	1732.99	{206., 5121.}	521.458	0.622062	newly 5 weeks spec	112.	127.23	{2., 318.}	157.313	0.953201	newly 5 weeks cumul	495.	2052.27	{208., 6843.}	331.945	0.468672	newly 47 weeks cumul	2645.	2289.79	{243., 8363.}	103.628	0.600433
18	newly 3 weeks spec	134.	280.15	{16., 897.}	163.44	0.425148	newly 3 weeks cumul	498.	3074.32	{333., 10 546.}	527.378	0.542002	newly 5 weeks spec	79.	23.11	{0., 62.}	75.9367	1.06121	newly 5 weeks cumul	686.	3175.49	{355., 10 713.}	378.652	0.461852	newly 47 weeks cumul	921.	3186.27	{355., 10 716.}	267.163	0.383482
19	newly 3 weeks spec	74.	69.13	{1., 111.}	136.419	0.968232	newly 3 weeks cumul	333.	606.03	{62., 828.}	201.48	0.621844	newly 5 weeks spec	49.	27.4	{0., 19.}	126.286	1.55712	newly 5 weeks cumul	449.	679.28	{63., 974.}	179.51	0.690912	newly 50 weeks cumul	666.	709.31	{63., 1038.}	146.59	0.8029
20	newly 3 weeks spec	165.	383.72	{12., 1152.}	221.515	0.686117	newly 3 weeks cumul	661.	2617.93	{256., 9654.}	337.902	0.457944	newly 5 weeks spec	141.	53.42	{0., 149.}	81.7872	1.26235	newly 5 weeks cumul	964.	2816.84	{257., 10 118.}	251.977	0.482001	newly 51 weeks cumul	1374.	2863.11	{257., 10 145.}	186.509	0.522316
21	newly 3 weeks spec	122.	265.81	{0., 1250.}	251.418	1.69335	newly 3 weeks cumul	466.	1570.33	{171., 4941.}	277.813	0.401371	newly 5 weeks spec	62.	92.14	{0., 120.}	202.097	1.71708	newly 5 weeks cumul	595.	1823.15	{175., 7813.}	264.516	0.446935	newly 51 weeks cumul	738.	1914.63	{177., 8512.}	234.362	0.495243
22	newly 3 weeks spec	145.	160.37	{0., 401.}	110.834	0.978888	newly 3 weeks cumul	458.	984.42	{244., 2128.}	140.812	0.292363	newly 5 weeks spec	86.	79.18	{0., 237.}	108.047	1.10935	newly 5 weeks cumul	631.	1178.93	{369., 2675.}	120.962	0.28153	newly 47 weeks cumul	1216.	1602.5	{378., 4448.}	105.16	0.395452
23	newly 3 weeks spec	51.	94.65	{0., 71.}	200.529	0.992541	newly 3 weeks cumul	384.	814.98	{173., 616.}	166.359	0.284021	newly 5 weeks spec	14.	11.82	{0., 21.}	127.	1.36035	newly 5 weeks cumul	436.	859.37	{174., 626.}	160.144	0.310902	newly 52 weeks cumul	442.	866.43	{174., 626.}	159.45	0.312758
24	newly 3 weeks spec	109.	121.58	{2., 200.}	140.642	0.827295	newly 3 weeks cumul	313.	708.64	{158., 1335.}	168.658	0.306835	newly 5 weeks spec	56.	28.63	{0., 83.}	105.339	1.58167	newly 5 weeks cumul	432.	796.68	{158., 1692.}	152.06	0.376696	newly 48 weeks cumul	566.	830.8	{158., 2151.}	134.286	0.44349
25	newly 3 weeks spec	134.	102.6	{0., 99.}	132.866	1.67766	newly 3 weeks cumul	550.	934.81	{36., 2667.}	162.638	0.59652	newly 5 weeks spec	58.	78.87	{0., 56.}	189.086	1.57088	newly 5 weeks cumul	716.	1108.88	{46., 4122.}	153.14	0.609482	newly 55 weeks cumul	798.	1329.8	{52., 4774.}	163.233	0.611152
26	newly 3 weeks spec	59.	177.24	{26., 195.}	229.831	0.421946	newly 3 weeks cumul	261.	1699.08	{452., 4297.}	554.927	0.591365	newly 5 weeks spec	33.	42.32	{0., 79.}	112.364	0.805231	newly 5 weeks cumul	353.	1819.27	{592., 5482.}	419.312	0.51243	newly 46 weeks cumul	475.	1910.75	{696., 5552.}	306.183	0.421916
27	newly 3 weeks spec	87.	206.63	{1., 777.}	223.828	0.796769	newly 3 weeks cumul	397.	1988.33	{373., 7311.}	409.373	0.384666	newly 5 weeks spec	24.	41.09	{0., 96.}	181.542	1.14359	newly 5 weeks cumul	477.	2117.04	{382., 8987.}	354.574	0.344861	newly 48 weeks cumul	499.	2190.47	{385., 9359.}	347.894	0.333149
28	newly 3 weeks spec	95.	14.38	{0., 36.}	87.1579	1.6013	newly 3 weeks cumul	273.	279.21	{6., 522.}	107.864	0.753066	newly 5 weeks spec	72.	2.86	{0., 10.}	96.0278	2.21568	newly 5 weeks cumul	428.	287.98	{6., 540.}	87.7477	0.837182	newly 26 weeks cumul	1195.	292.19	{6., 542.}	87.641	1.21404
29	newly 3 weeks spec	140.	182.95	{0., 213.}	148.007	1.47612	newly 3 weeks cumul	520.	1409.81	{431., 1837.}	187.867	0.269409	newly 5 weeks spec	86.	80.13	{0., 151.}	134.174	1.57137	newly 5 weeks cumul	706.	1613.8	{495., 2869.}	160.164	0.267259	newly 49 weeks cumul	1008.	1825.78	{515., 4115.}	136.917	0.311342
30	newly 3 weeks spec	99.	116.04	{0., 217.}	128.586	1.52175	newly 3 weeks cumul	453.	1462.77	{393., 2723.}	230.898	0.357713	newly 5 weeks spec	51.	41.89	{0., 77.}	125.51	1.56004	newly 5 weeks cumul	566.	1569.9	{503., 2842.}	185.276	0.293806	newly 48 weeks cumul	783.	1668.67	{673., 2864.}	127.544	0.218465
31	newly 3 weeks spec	92.	117.72	{0., 111.}	163.13	1.49655	newly 3 weeks cumul	415.	960.03	{36., 1934.}	202.475	0.560817	newly 5 weeks spec	49.	59.78	{0., 63.}	166.857	1.48263	newly 5 weeks cumul	552.	1109.05	{52., 2237.}	178.607	0.559941	newly 55 weeks cumul	668.	1234.03	{59., 2355.}	166.531	0.572419
32	newly 3 weeks spec	124.	32.69	{0., 68.}	82.3145	1.31916	newly 3 weeks cumul	386.	600.26	{159., 768.}	75.6321	0.245446	newly 5 weeks spec	137.	6.43	{0., 19.}	96.4307	2.31198	newly 5 weeks cumul	648.	619.82	{203., 810.}	54.1975	0.272557	newly 28 weeks cumul	1971.	629.27	{334., 818.}	78.0015	0.686343
33	newly 3 weeks spec	96.	201.45	{0., 769.}	211.698	1.36804	newly 3 weeks cumul	537.	2044.28	{389., 6091.}	301.777	0.346486	newly 5 weeks spec	49.	95.53	{0., 360.}	212.061	1.42493	newly 5 weeks cumul	666.	2275.79	{432., 8273.}	270.86	0.354691	newly 49 weeks cumul	775.	2576.22	{450., 8507.}	267.804	0.376712
34	newly 3 weeks spec	217.	230.08	{0., 667.}	133.373	1.31633	newly 3 weeks cumul	685.	1951.4	{420., 5430.}	215.971	0.307553	newly 5 weeks spec	140.	93.41	{0., 191.}	112.207	1.60714	newly 5 weeks cumul	982.	2190.12	{466., 5920.}	173.226	0.321865	newly 49 weeks cumul	1514.	2411.83	{488., 7583.}	139.224	0.410269
35	newly 3 weeks spec	115.	253.06	{0., 659.}	221.965	1.46412	newly 3 weeks cumul	444.	1929.46	{452., 5526.}	339.775	0.398556	newly 5 weeks spec	59.	126.03	{0., 494.}	230.898	1.44563	newly 5 weeks cumul	616.	2233.11	{520., 7757.}	276.255	0.356351	newly 49 weeks cumul	837.	2586.45	{547., 9888.}	238.152	0.357109
36	newly 3 weeks spec	124.	17.02	{0., 43.}	91.871	2.36675	newly 3 weeks cumul	489.	1446.57	{47., 5030.}	242.607	0.512234	newly 5 weeks spec	43.	11.77	{0., 28.}	97.9302	2.03485	newly 5 weeks cumul	588.	1473.04	{65., 5030.}	196.976	0.461575	newly 51 weeks cumul	620.	1574.1	{78., 5030.}	195.41	0.443345
37	newly 3 weeks spec	61.	273.11	{33., 185.}	379.656	0.311021	newly 3 weeks cumul	281.	851.46	{172., 638.}	234.555	0.276131	newly 5 weeks spec	9.	154.91	{12., 155.}	1626.78	0.687619	newly 5 weeks cumul	307.	1228.66	{224., 939.}	318.495	0.277017	newly 54 weeks cumul	480.	1710.22	{319., 2645.}	284.333	0.302394
38	newly 3 weeks spec	169.	230.69	{0., 726.}	163.024	1.22045	newly 3 weeks cumul	582.	2528.31	{72., 8737.}	397.593	0.600108	newly 5 weeks spec	127.	22.5	{0., 84.}	86.0472	1.89651	newly 5 weeks cumul	845.	2618.08	{78., 9304.}	290.947	0.642269	newly 50 weeks cumul	1549.	2630.86	{79., 9316.}	175.002	0.730728
39	newly 3 weeks spec	23.	44.45	{0., 92.}	217.783	1.54096	newly 3 weeks cumul	145.	1573.97	{54., 4500.}	1007.46	0.708752	newly 5 weeks spec	14.	50.03	{0., 59.}	384.643	1.44631	newly 5 weeks cumul	176.	1673.27	{91., 4500.}	870.915	0.680205	newly 51 weeks cumul	182.	1985.71	{112., 6822.}	1007.62	0.724902
40	newly 3 weeks spec	69.	506.29	{0., 2692.}	708.71	1.48197	newly 3 weeks cumul	223.	2807.05	{670., 9878.}	1162.14	0.851665	newly 5 weeks spec	71.	261.48	{0., 1070.}	343.972	1.37352	newly 5 weeks cumul	369.	3431.84	{873., 12 858.}	833.469	0.729286	newly 24 weeks cumul	1107.	4106.53	{1069., 14 699.}	275.585	0.354082
41	newly 3 weeks spec	7.	220.49	{0., 257.}	3093.	1.07374	newly 3 weeks cumul	98.	1006.3	{20., 2920.}	950.041	0.756434	newly 5 weeks spec	0.1	116.34	{0., 304.}	116 298.	1.70325	newly 5 weeks cumul	102.	1289.9	{21., 3267.}	1187.35	0.805741	newly 55 weeks cumul	102.	1525.64	{24., 3267.}	1418.06	0.850637
42	newly 3 weeks spec	113.	88.21	{0., 138.}	90.2035	1.23786	newly 3 weeks cumul	500.	1334.48	{129., 3058.}	196.836	0.40927	newly 5 weeks spec	90.	31.49	{0., 43.}	96.2111	1.58745	newly 5 weeks cumul	679.	1418.43	{157., 3080.}	142.255	0.35086	newly 47 weeks cumul	908.	1488.79	{170., 3247.}	105.552	0.319769
43	newly 3 weeks spec	16.	81.64	{0., 29.}	535.625	1.25189	newly 3 weeks cumul	172.	694.43	{74., 798.}	332.157	0.439434	newly 5 weeks spec	1.	13.79	{0., 2.}	1437.	0.953257	newly 5 weeks cumul	187.	744.3	{74., 798.}	329.123	0.432682	newly 53 weeks cumul	187.	752.9	{74., 798.}	333.722	0.433799
44	newly 3 weeks spec	169.	131.63	{2., 133.}	106.964	0.829667	newly 3 weeks cumul	606.	1755.68	{304., 6496.}	241.594	0.359961	newly 5 weeks spec	127.	98.73	{0., 46.}	141.693	1.52839	newly 5 weeks cumul	896.	1966.71	{331., 6496.}	194.749	0.433887	newly 49 weeks cumul	1535.	2151.02	{340., 7521.}	144.42	0.549152
45	newly 3 weeks spec	53.	135.2	{12., 219.}	205.472	0.37375	newly 3 weeks cumul	403.	987.07	{257., 1197.}	175.809	0.226669	newly 5 weeks spec	9.	49.81	{0., 70.}	508.556	0.679228	newly 5 weeks cumul	435.	1117.12	{278., 1324.}	187.453	0.240852	newly 56 weeks cumul	452.	1190.39	{284., 1538.}	194.914	0.252583
46	newly 3 weeks spec	126.	131.42	{0., 194.}	135.714	1.13297	newly 3 weeks cumul	521.	636.07	{41., 858.}	120.616	0.550003	newly 5 weeks spec	38.	104.96	{0., 231.}	293.526	1.2777	newly 5 weeks cumul	617.	856.56	{46., 1636.}	133.436	0.546794	newly 53 weeks cumul	658.	1520.58	{53., 5396.}	217.225	0.592024
47	newly 3 weeks spec	195.	68.47	{0., 104.}	91.6667	1.70289	newly 3 weeks cumul	736.	619.31	{64., 1006.}	88.091	0.562958	newly 5 weeks spec	94.	62.08	{0., 130.}	113.617	1.70762	newly 5 weeks cumul	971.	747.52	{84., 1630.}	86.3213	0.586659	newly 53 weeks cumul	1232.	1293.7	{107., 4757.}	112.287	0.633027
48	newly 3 weeks spec	116.	202.45	{7., 441.}	202.784	0.768986	newly 3 weeks cumul	481.	956.61	{116., 2512.}	187.881	0.484332	newly 5 weeks spec	50.	72.38	{0., 73.}	192.12	1.14068	newly 5 weeks cumul	630.	1148.46	{124., 2760.}	181.844	0.530966	newly 50 weeks cumul	758.	1235.81	{124., 2837.}	170.674	0.571026
49	newly 3 weeks spec	138.	87.13	{0., 157.}	83.1957	1.04433	newly 3 weeks cumul	440.	510.32	{97., 769.}	62.1909	0.263276	newly 5 weeks spec	140.	52.43	{0., 184.}	84.9643	1.32199	newly 5 weeks cumul	714.	627.85	{122., 910.}	66.0938	0.351317	newly 46 weeks cumul	1855.	928.88	{179., 2868.}	73.4631	0.59031
50	newly 3 weeks spec	69.	187.36	{22., 444.}	220.	0.326875	newly 3 weeks cumul	243.	647.44	{122., 1144.}	185.638	0.249491	newly 5 weeks spec	43.	116.67	{5., 249.}	248.302	0.53668	newly 5 weeks cumul	357.	912.8	{164., 2328.}	185.221	0.259001	newly 46 weeks cumul	518.	1321.76	{230., 4736.}	201.726	0.325982
